# A User’s Guide to Open Educational Resources in Medical Education

**DOI:** 10.7759/cureus.93394

**Published:** 2025-09-28

**Authors:** Teresa M Chan, N Seth Trueger, Lauren A Maggio, Daniel K Ting, Jonathan Sherbino, Brent Thoma

**Affiliations:** 1 School of Medicine, Toronto Metropolitan University, Toronto, CAN; 2 Department of Emergency Medicine, Northwestern University Feinberg School of Medicine, Chicago, USA; 3 Department of Medical Education, University of Illinois at Chicago, Chicago, USA; 4 Department of Emergency Medicine, University of British Columbia, Kelowna, CAN; 5 Department of Medicine, McMaster University, Hamilton, CAN; 6 Department of Emergency Medicine, University of Saskatchewan, Saskatoon, CAN

**Keywords:** critical appraisal, free open access medical education, online medical education, open educational resources, users guide

## Abstract

Open educational resources (OERs) such as blog posts, podcasts, infographics, and videos focusing on medical topics are frequently published online. Their objectives are variable and include the critical appraisal of individual research articles, the knowledge translation of new or under-discussed publications or guidelines, and the review and integration of knowledge on a particular topic. However, due to the ease of publishing in these new media, the quality of these resources is heterogeneous and inconsistent. It is important for medical learners, educators, and practicing physicians to critically appraise these new and easily accessible formats of medical literature and resources. This paper provides an approach to appraise and use OERs.

## Editorial

Clinical scenario

A patient presents to the emergency department in respiratory distress. As the healthcare team makes plans to intubate the patient, a resident team member, referencing a podcast, suggests providing the patient with apneic oxygenation via a high-flow nasal cannula. The podcast suggested this was a successful treatment used to prevent peri-intubation desaturation. The respiratory therapist indicates that they read similar information on a blog post. The team ponders how best to handle these new sources of rapid medical knowledge dissemination.

Introduction

In 2023 alone, the National Library of Medicine indexed 1,279,327 articles for inclusion in Medline [1]. Navigating this amount of new evidence for application into practice is a considerable challenge for clinicians. An estimated average number-needed-to-read for top clinical journals is 14, meaning a clinician would need to read 14 articles before identifying a high-quality, clinically relevant article [2]. This is the challenge of knowledge translation: the synthesis, dissemination, exchange, and application of knowledge to improve health and healthcare [3]. It is not easy to find relevant articles: search terms are difficult to navigate, especially with a just-in-time search [4], and once a relevant article is found, access may be restricted by paywalls. Clinician uptake and use of systematic reviews is poor [5]. Finally, the peer review and publication process turnaround time can inhibit the timely distribution of information [6]. These challenges have precipitated the rise of second-order peer review: resources that concisely report the findings of original research. Institutional or commercial second-order peer review sources are available [7]. However, significant institutional resources or high subscription fees are required to maintain them.

The challenges of traditional publishing can lead to delays in health research translation. The process of knowledge translation from scientific discovery to application in clinical practice has been estimated to take 17 years [8]. In contrast, digital media (particularly the internet, social media) has democratized the near-instantaneous delivery of medical information in a connected world. The ability to easily access or review information, either just-in-time in the clinical environment or while engaging in other activities such as exercising or commuting, is one of the drivers of the open educational resource (OER) movement [9]. OERs serve a similar function to second-order traditional reviews: identifying and condensing key messages from the primary literature. While the number of resources published in the grey literature poses the same challenge as the indexed literature, search engine algorithms have partially solved issues of identifying relevant and accessible resources.

For healthcare providers, the rise of the Free Open Access Medical (FOAM) education movement has allowed for instantaneous sharing of new evidence through OERs such as blogs, podcasts, and infographics [9-14]. The grassroots FOAM movement has encouraged many medical educators to contribute freely accessible [11] OERs via various social platforms, including blogs, podcasts, social media platforms BlueSky, X, Instagram, and TikTok, leading to an unprecedented rise in the number of OERs available to health professionals [10]. These resources allow clinicians to quickly access information in simple formats that can be applied in the clinical setting [15]. They frequently align with the lifestyle habits of the learner in the continuing education space, which helps to enhance their versatility (e.g., listening to podcasts while commuting, reviewing an infographic on one’s phone while waiting in line), and can be integrated into formal curricula (e.g. assigning a podcast for home listening prior to a traditional in-person morning report or conference).

OERs are created by individuals, consortia, and institutions, and vary in their focus and the support their creators receive for their production. Some are individually funded by content producers, while others may be supported by residency programs, research groups, or larger institutions and organizations (including businesses). Stylistically, OERs vary in their level of formality and balance between opinion-focused tacit knowledge and evidence-informed analysis, as well as the depth of their content (i.e., deep dives vs. summaries). While many OERs have no formal creation or oversight processes, some are becoming increasingly sophisticated in their use of prepublication peer review and editorial oversight [16-19]. Table 1 provides an overview that defines the ideal path for health evidence to become an OER.

**Table 1:** Process that defines the ideal path of evidence to develop an OER OER: open educational resource

Steps in the process
1. Evidence is generated.
2. A need for the dissemination of this evidence is identified within a field.
3. Resources are selected and aggregated to formulate an OER.
4. The best knowledge dissemination strategy is determined.
5. Content is generated by resources that are aligned with the best strategy.
6. Pre-publication editorial or peer review is conducted.
7. The resource is published openly.
8. The resource is disseminated via various social media platforms.
9. Post-publication critique of the resource is conducted.
10. Revisions are made and openly acknowledged with versioning and archival of amendments.

Unfortunately, OERs are not consistently high quality [20,21]. Although it facilitates the ease of publication and generation, the nature and ease of internet publishing mean there are no inherent, required quality controls in comparison to traditional publications. Popularity or audience size (e.g., web traffic, subscribers, downloads) may serve as intuitive surrogates for quality (e.g., selecting the OER at the top of the search or with the most followers), but can be gamed by publishers via search engine optimization. This article provides a systematic approach to critically appraising OERs for application to clinical practice.

How to find OERs

General web search engines are a common method to locate OERs. Most search engines track their users' use of the pages they are directed to and use this information to improve the accuracy and quality of search results related to the keywords that were used to find them. Generally, search functions yield frequently accessed resources where users spend more time. Customized academic, medical, and OER search engines have been developed for the unique needs of healthcare professionals, as opposed to the general search engines created for the public [22]. Table 2 summarizes various ways to find OERs.

**Table 2:** Finding and accessing OERs OER: open educational resource

Method of accessing OERs	Explanation	Examples
Digital platform repository	Search common repositories of various digital platforms (e.g., podcasts, videos, etc.)	YouTube, Vimeo, Libsyn, Soundcloud
Direct website access	Directly access a website that archives and collates OERs. Search within the website.	Life in the Fast Lane, Academic Life in Emergency Medicine, OERCommons, MERLOT
Email newsletters	Subscribe to the newsletters of producers of OERs for passive delivery of content.	EMCrit (see https://emcrit.org/subscribe/)
Search engines (including specialized & customized ones)	Use general search engines to identify relevant OERs, including searching for videos and images.	Google, Bing, Google Scholar, Google FOAM
Social media sharing	Directly access OERs recommended on social media. Use hashtags to refine the recommended content.	X, BlueSky, Facebook, Instagram, TikTok
Subscription	Subscribe to an OER (e.g., YouTube channel, podcast, blog, etc.) for regular passive delivery of content.	JAMA Network Multimedia

Imperative to critically appraise OERs

The rapid dissemination of online education also presents a threat. In the absence of a critical review, rapid dissemination of and easy access to information risks spreading misinformation. This imperative is especially important for new and emerging topics. The thrill or reward of being the first to publish an OER on a new emerging therapy or clinical decision tool may lead OER producers towards skewing their content (and therefore their audience) towards these early reports, rather than providing fulsome systematic approaches to generating content [20,23]. In many instances, initial studies of an intervention have been promising, but later studies nullify or contradict those results [24-26]. There are multiple reasons for this phenomenon; initial studies involving smaller numbers of participants will emerge quickly and are often wrought with publication bias [27]. OERs reviewing the latest evidence from individual studies should be reviewed with particular caution, as it often takes time and further knowledge synthesis or meta-analyses to clarify the overall state of the evidence.

Tools to evaluate OERs

Research on OER use in education and knowledge translation is increasing [28]. There are three key areas to consider when evaluating OERs: content, credibility, and design [29,30]. Twelve decision tools and checklists to review and appraise the quality of OERs have been published [21,30-47]. Two scores stand out with the strongest validity evidence: the Academic Life in Emergency Medicine (ALiEM) Approved Instructional Resources (AIR) score [36-38] and the Medical Education Translational Resources: Impact and Quality (METRIQ) scores [39-41]. These evaluation tools, alongside the other frameworks and literature [32-35] from a recent rapid systematic review [21], inform the recommendations below. Table 3 describes the 11 tools and checklists to evaluate the quality of OERs.

**Table 3:** Tools for evaluating the quality of OERs ALiEM: Academic Life in Emergency Medicine; AIR: Approved Instructional Resources [36-38,46]; CCMEWQET: Critical Care Medical Education Website Quality Evaluation Tool [34]; DIF: Digital Impact Factor [47]; METRIQ: Medical Education Translational Resources Impact and Quality [39,40]; MEWQET: Medical Education Website Quality Evaluation Tool [32]; MEOW: Modified Education in Otolaryngology Website assessment tool [33]; SMi: Social Media Index [40,44]; rAIR: revised Approved Instructional Resources [38], rMETRIQ: revised Medical Education Translational Resources Impact and Quality [41]; OER: open educational resource; Quality Checklist for Blogs and Podcasts [31]

Name	Type of scoring tool	Intended use	Domains assessed	Limitations	Evidence
ALiEM AIR [36-38,46]	Individual resources (e.g., blog post, podcast episode)	Educators in emergency medicine	1. Impact on emergency medicine clinical practice; 2. Content accuracy; 3. Educational utility; 4. Evidence-based medicine; 5. Referencing	Expert judgment required to score. Rater training and calibration required. Original components lack strong content validity.	Broad validation across a heterogeneous group of users in study comparing METRIQ-8 [39,40] to the ALiEM AIR scoring tool [37-38] and the Social Media Index [40,44].
Critical Care Medical Education Website Quality Evaluation Tool (CCMEWQET) [34]	Entire OER website (e.g., entire blog or podcast)	Educators in critical care	1. Authorship, credibility, and disclosure; 2. Aim, scope, and intended audience; 3. Content quality; 4. Currency of information; 5. Content; 6. Navigability and speed; 7. Access; 8. Interactivity; 9. Graphics and media; 10. Layout and design; 11. Hyperlinks	Assessors of websites were not blinded to the identity of the website during the original study. No reliability scoring performed.	Only a derivation study has been done.
Digital Impact Factor (DIF) [47]	Entire OER website (e.g., entire blog or podcast)	Healthcare provider of any level (from trainee to faculty member), authors, researchers, promotion and tenure committees	1. Ahrefs Domain Rating; 2. Twitter/X; 3. Facebook; 4. Instagram; 5. YouTube and Vimeo; 6. TikTok; 7. LinkedIn; 8. Reddit; and 9. Pinterest	Websites have varying levels of quality for each post as there are often many authors and the editorial oversight may be inconsistent. May disproportionately reward website authors who have a strong social media presence.	Validation within emergency medicine compared to the Social Media Index [47].
Gestalt score [42,43]	Individual resources (e.g., blog post, podcast episode)	Healthcare provider of any level (from trainee to faculty member)	Overall Gestalt (gut feeling)	Need many independent raters to achieve good reliability. Non-standardized, based on individual idiosyncrasy/bias.	Developers have attempted broad validation, but the reliability of the scoring tool is inconsistent [42,43].
METRIQ-5 score [39]	Individual resources (e.g., blog post, podcast episode)	Originally derived to assist inexperienced users (e.g., junior learners)	1. Concise content; 2. References; 3. Background; 4. Moderation; 5. Publisher (Eliminates 3 items from METRIQ-8)	Similar to METRIQ-8 tool.	Only a derivation study has been done.
METRIQ-8 score [39,40]	Individual resources (e.g., blog post, podcast episode)	Originally derived to assist inexperienced users (e.g., junior learners)	1. Concise content; 2. Content construction; 3. References; 4. Editorial Process; 5. Consistency with citations; 6. Background; 7. Moderation; 8. Publisher	Has similar performance to the Gestalt score; as such has limited use in those with advanced training (e.g., faculty members, attendings).	Broad validation across a heterogeneous group of users in study comparing METRIQ-8 [39,40] to the ALiEM AIR scoring tool [37,40] and the Social Media Index [40].
Quality Checklist for Blogs and Podcasts [31]	Individual resources (e.g., blog post, podcast episode)	Healthcare provider of any level (from trainee to faculty member)	1. Credibility; 2. Content; 3. Design	Rating using dichotomous criteria can be difficult for junior or inexperienced user. Overall scoring and weighting of the checklist is inexplicit.	Only a derivation study has been done.
Medical Educational Website Quality Evaluation Tool (MEWQET) [32]	Entire OER website (e.g., entire blog or podcast)	Educators in pathology	1. Authorship and credibility' 2. Aim, scope and intended audience; 3. Comprehensiveness; 4. Currency of information; 5. Content; 6. Navigability; 7. Speed; 8. Access; 9. Hyperlinks; 10. Graphics and design; 11. Interactivity; 12. Disclosures	Assessors of websites were not blinded to the identity of the website during the original study. Score does not correlate with Google PageRank and Alexa Traffic Rank, which suggests a lack of external validity.	Narrow validation of the tool against the Gestalt opinion of two expert pathologists.
Modified Education in Otolaryngology Website (MEOW) assessment tool [33]	Entire OER website (e.g., entire blog or podcast)	Educators in otolaryngology	1. Authorship, credibility, and disclosure; 2. Frequency of revision; 3. Content quality; 4. Interactivity;5. Graphic elements and media; 6. Layout and design; 7. Navigability and speed; 8. Hyperlinks	Assessors of websites were not blinded to the identity of the website during original study.	Developers have attempted narrow validation during the initial study, although the scores only partially correlated with the gold standard (blinded expert otolaryngologist rater).
Revised AliEM AIR (rAIR) tool [38]	Individual resources (e.g., blog post, podcast episode)	Educators in emergency medicine	1. Impact on emergency medicine clinical practice; 2. Content accuracy; 3. Educational utility; 4. Evidence-based medicine; 5. Referencing; 6. Author credibility	Based on the AliEM AIR tool. Refined using blog articles only.	The derivation study included pilot testing of the tool [38].
Revised METRIQ (rMETRIQ) score [41]	Individual resources (e.g., blog post, podcast episode)	A revision of the METRIQ-8 scoring tool based on end-user feedback. Meant for healthcare provider of any level (from trainee to faculty member)	1. Concise content; 2. Content construction and editorial process; 3. References; 4. Background; 5. Publisher; 6. Writing quality; 7. Post-publication commentary	Based on the METRIQ-8 score. More sophisticated than prior iterations and refined using blog articles only.	Only a derivation study has been done [41].
Social Media Index (SMi) [40,44]	Entire OER website (e.g., entire blog or podcast)	Healthcare provider of any level (from trainee to faculty member) in emergency medicine and/or critical care	1. Alexa website ranking; 2. Twitter/X; 3. Facebook	Websites have varying levels of quality for each post as there are often many authors and the editorial oversight may be inconsistent. May disproportionately reward website authors who have a strong social media presence.	Broad validation of the entire index within emergency medicine compared to specialty journals [44], and also compared to the ALiEM AIR and METRIQ-8 scores for randomly selected posts [40].

The tools are variable in their derivation, validation, scope, style, and purpose. For example, Gestalt evaluation (i.e., general, non-systematic, non-referenced impression by the reader) has poor reliability [43,45]. In contrast, the METRIQ scores were derived via a multi-study process [37,39,41,44]. The validity evidence for the Social Media Index (SMi), AIR tool, and METRIQ score has been evaluated within emergency medicine with mixed results [40,46]. All of the website-focused tools were developed within the context of particular medical specialties (emergency medicine, critical care, pathology, and otolaryngology) and have not been evaluated in broader contexts. Some take a checklist approach (e.g., Quality Checklist for Blog and Podcasts), others rate items on anchored scales (e.g., METRIQ scores, ALiEM AIR tool), and one (the Digital Impact Factor) uses purely quantitative methods to evaluate the impact and followership of OER websites.

Commonalities across tools include items related to the evaluation of the OER producers’ credibility, the disclosure of conflicts of interest [35], the description of editorial or review processes, and referencing supporting primary research. While most tools were developed for the end users, the ALiEM AIR tool was developed to help teachers select resources for learners [36,37,46]. This approach resulted in the inclusion of items uncommon in critical appraisal tools, such as the presence of educational pearls and the impact on practice. Furthermore, several tools (e.g., Modified Education in Otolaryngology Website (MEOW) assessment tool, Critical Care Medical Education Website Quality Evaluation Tool (CCMEWQET), Quality Checklists for Blogs and Podcasts) went beyond critical appraisal of the content to evaluate the design, interactivity, and usability of the digital resources. While irrelevant to the accuracy of the information presented, these features are arguably important given the educational and/or translational intent of these resources.

How to appraise OERs

Instead of simply endorsing the best tools forward, we have sought to reconcile the elements of these tools via our own synthesis. While many of which have robust derivation processes and validity evidence, when teaching the skill of critical appraisal we acknowledge that it may be important to go further than simply guiding learner via surface features of websites (e.g., as the METRIQ scores do [39,41]) or completely trusting an educator’s Gestalt (e.g., Gestalt [39,42] or ALiEM AIR [36-38]). Table 4 features five key questions that trainees, clinicians, or teachers might use in selecting high-quality OERs.

**Table 4:** Five key questions to address in the evaluation of OERs OER: open educational resource

Key questions
1. Can you identify the OER’s creator(s)?
2. Is the creator(s) of the OER credible (i.e., Do they have their content expertise? Do they have conflicts of interest?)?
3. Does the OER cite supporting primary references and peer-reviewed citations?
4. Is the OER clear and organized?
5. Is there a formal editorial process that occurs before (peer review, editorial process) or after publication (e.g., responsiveness to comments)?

*Can You Identify the OER’s Creator(s)?* 

The degree to which the creators of OERs are identifiable is variable. Some OERs are attributable to identifiable individuals with listed credentials with readily identifiable online footprints, while others use pseudonyms or are published anonymously. Resources that are unattributed or poorly attributed are suspect because no one stands behind their veracity.

Is the Creator(s) of the OER Credible?

Do the author(s) have content experience, as suggested by training, or appropriate practice in the domain? Does the experience match the complexity of the topic? For example, resources directed towards a novice clinician may be best produced by a clinician with a close sense of the challenges of the novice clinician and not necessarily by a senior clinician far removed from the experience of a novice. Equally, content addressing complex or emerging knowledge may be best produced by experts in the discipline. Is there a conflict of interest, such as financial associations with the content (i.e., patents pending, personal investments) or intellectual interests in a particular stance on a topic? While the expertise of the author may be obvious, is the content misrepresented by a financial or proprietary interest? Is there a statement of conflict of interest? The absence of such a statement is concerning. 

Does the OER Cite Supporting Primary References and Peer-Reviewed Studies?

Generally, OER creators should cite the primary references and peer-reviewed studies that support their content. Are these citations from peer-reviewed, indexed journals? A lack of citations or the citation of only secondary resources is suspect. Are the citations mainly self-citations? Extensive self-citation concerning as it may result in the unbalanced representation of the topic. If the topic is established, do the creators cite any synthetic reviews? OERs that do not stand on a balanced representation of the theory, research, and literature that bounds the topic are suspect. Lastly, are the references cited alongside specific information (as opposed to a list of references at the end of a resource). The integration of in-line citations allows the references supporting specific information to be reviewed.

Is the OER Clear and Organized?

The design of an OER is irrelevant to the quality of the information it presents, but it can preclude effective knowledge uptake by readers. Educators know that the impact and clarity of an OER are highly dependent on design. Critical appraisal of the design of an educational resource is appropriate to ensure an accurate message is being conveyed.

Clarity of presentation (writing, sound mixing, cinematography) is important for conveying one’s message; this is especially so when aggregating or interpreting the work of others in a secondary resource. Mayer’s twelve principles on multimedia learning highlight elements of text, voice, image, and animation use in a multimedia resource [48,49]. Those elements should complement each other, while the redundant information should be eliminated to decrease cognitive load [48,50]. Short-term learning is highly affected by the better design of elements [49], which increases learners’ satisfaction and motivation for the next learning experience [51]. Complexity of the content and heavy use of visual materials can create cognitive load, which may not achieve transfer of knowledge to readers. Interactive materials can increase the use of OERs [52]. Lastly, learners access the resources with different devices and platforms. Responsive design supports every screen size, which results in better turn-out on learning [53].

Is There a Formal Editorial Process That Occurs Before or After Publication?

There are several ways in which these non-traditional publishing methods allow for producers to optimize their OER content. Readers should examine OERs for evidence of an editorial process such as pre-publication review by editors and/or peer reviewers. Some FOAM blogs incorporate a layer of editorial review [19] or peer review into their pre-publication processes [16,17]. Others harness the power of the social aspects of these new media to integrate post-publication peer review. This is often seen in the comments sections of the OER platforms that allow for discussion of the resource between its creators and users. Resources with comments from users that are responded to effectively by the creator can add additional context to the resource and support its credibility. If errors or omissions are identified by users, their correction also enhances credibility. Ideally, changes made to the resource resulting from such interaction should be identifiable in some way [19].

Worked examples

**Figure 1:**
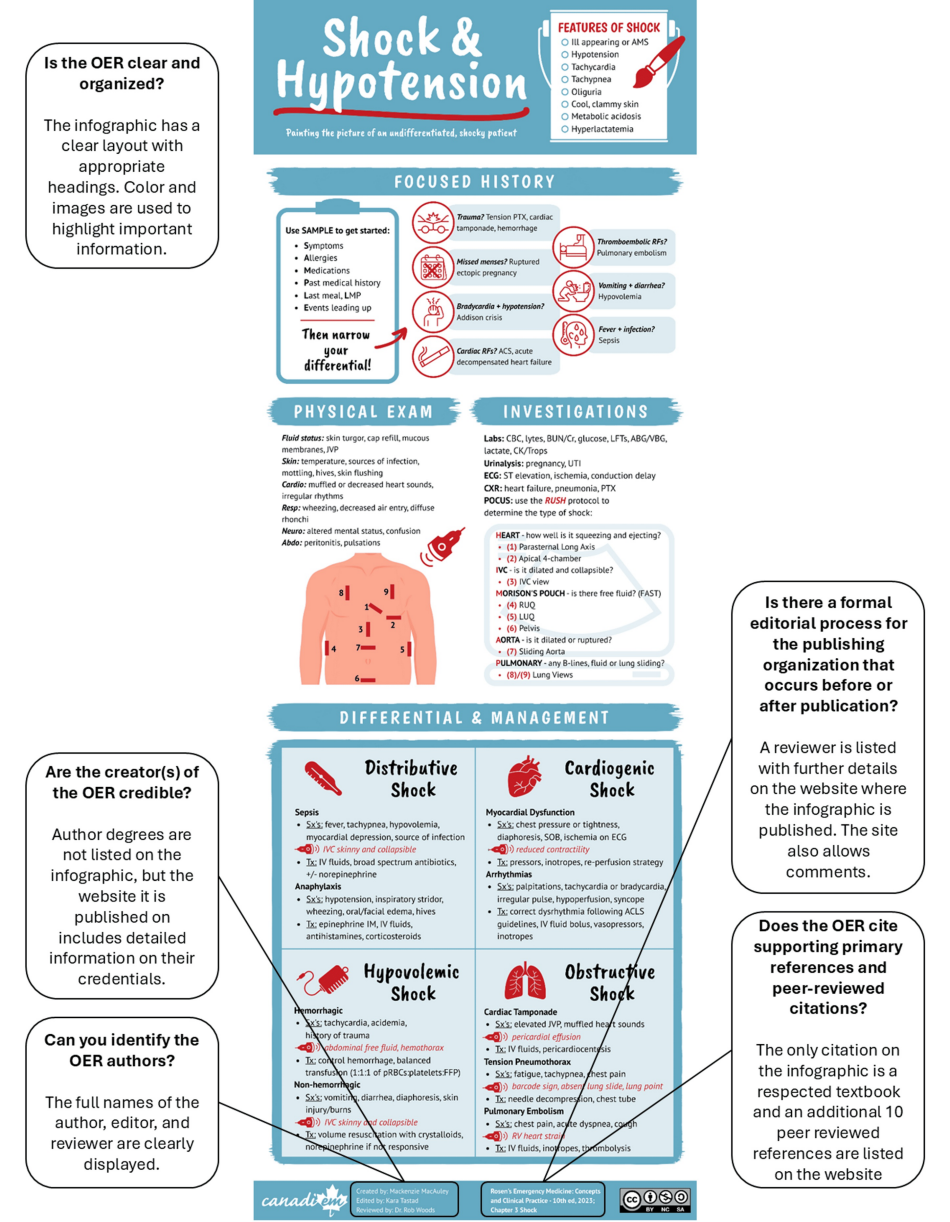
Using the guide to critically appraise an infographic on shock and hypotension This infographic was published under an Open Access license on the CanadiEM website [54]. OER: open educational resource

For the purposes of this guide, a pragmatic synthesis of existing tools was performed to provide general guidance for OER appraisal. Worked examples of how the guide can be used to evaluate different resources are found in Figure 1 and Table 5. 

**Table 5:** Using the guide to critically appraise an blog post discussing the CRASH3 study The reviewed blog post [55] and CRASH3 trial [56] are cited within the table. CRASH3: Corticosteroid Randomisation after Acute Severe Head injury – 3

Question	Critical appraisal
Blog post title	Tranexamic acid for traumatic brain injury (CRASH3) [54,55]
Blog post abstract	The CRASH-3 trial was a multicenter randomized controlled trial that enrolled approximately 13000 adult patients who suffered traumatic brain injury within 3 hours. Patients received either tranexamic acid or placebo with a primary outcome of head injury-related death within 28 days. The primary outcome was not statistically significant. However, the study authors did subgroup analyses that suggested that patients with moderate head injury may benefit from tranexamic acid. In this blog post, Dr. Josh Farkas argues that the results from this moderately injured subgroup make logical sense, are based on a large number of patients, and have good safety endpoints. Therefore, Dr. Farkas argues that the results from this trial are practice-changing and that clinicians should administer tranexamic acid in patients with moderate head injury.
Can you identify the OER’s creator(s)?	The author of this post, Josh Farkas, is clearly denoted at the bottom of the post. Dr. Farkas has a well-established digital identity and is easily found on search engines.
Is the creator(s) of the OER credible (i.e., Do they have their content expertise? Do they have conflicts of interest?)	Dr. Farkas lists a biography on the website that explains his basic and specialty medical training and his university affiliation. Furthermore, it is explicitly stated that he does not have any intellectual or financial conflicts of interest.
Does the OER cite supporting primary references and peer-reviewed citations?	In this post, five peer-reviewed references are provided that map to and support the statements in the article. The medical journals referenced are all indexed on Medline. The references of primary literature help to contextualize the background and need for the CRASH-3 study. Although no meta-analyses are cited, a meta-analysis was done by the authors of the CRASH-3 study to provide an overview of the state of the evidence, and this figure is displayed in the blog post. As an additional bonus, 16 links are provided at the bottom of the post that link the reader to other OERs on the topic, which provides an opportunity for self-directed learning. There are no missing references regarding statements of fact that are not common knowledge.
Is the OER clear and organized?	The blog post is written in a serious but conversational and succinct style that is straightforward to read. The post is divided into short segments that are signalled with clear headings (e.g., preamble, main results, summary, etc.). The summary section is listed in bullet points that help to emphasize the take-home messages. The post also includes a simple graphic and tables that summarize key data. These visual elements adapt well to different sized screens on a web browser or mobile device.
Is there a formal editorial process that occurs before (peer review, editorial process) or after publication (e.g. responsiveness to comments)?	The blog site (PulmCrit) does not have a formal pre-editorial process. There is a robust system for readers to provide comments with a request to include full name, credentials, and conflicts of interest. In this specific post, there is one comment that did not receive an author response.

Resolving the clinical scenario

The patient was successfully intubated without desaturation. After rounds, the attending physician pulled up the blog post and the podcast show notes. Team members appraised the resources using the questions from this guide. The attending physician added her Gestalt assessment that both resources were well constructed and balanced. Together, the team determined that these resources were credible, well-constructed, and useful. One had even been updated to discuss some of the contradictory findings in the literature on this topic. The healthcare team resolved to incorporate critical appraisal of OERs into their daily rounds, in a similar manner to how they currently review traditional publications.

Conclusion

The era of evidence-based medicine has informed a generation of clinicians to scrutinize primary research, yet too frequently, OERs are entering clinical conversations without the same rigorous attention to the quality of the information. While OERs are increasingly created and used in medical education and clinical practice, ease of access and audience size are not surrogates for quality. Systematic critical appraisal is required to identify high-quality resources. Clinicians and educators should incorporate the critical appraisal of OERs into existing or similar processes that teach critical appraisal of primary research. This article provides a five-question guide to scaffold the evaluation of digital secondary resources such as blogs, podcasts, infographics, or videos.

## References

[1] (2025). MEDLINE PubMed production statistics. https://www.nlm.nih.gov/bsd/medline_pubmed_production_stats.html.

[2] McKibbon KA, Wilczynski NL, Haynes RB (2004). What do evidence-based secondary journals tell us about the publication of clinically important articles in primary healthcare journals?. BMC Med.

[3] Straus SE, Tetroe J, Graham I (2009). Defining knowledge translation. CMAJ.

[4] Shariff SZ, Sontrop JM, Haynes RB (2012). Impact of PubMed search filters on the retrieval of evidence by physicians. CMAJ.

[5] Perrier L, Mrklas K, Shepperd S, Dobbins M, McKibbon KA, Straus SE (2011). Interventions encouraging the use of systematic reviews in clinical decision-making: a systematic review. J Gen Intern Med.

[6] Powell K (2016). Does it take too long to publish research?. Nature.

[7] Haynes RB, Cotoi C, Holland J (2006). Second-order peer review of the medical literature for clinical practitioners. JAMA.

[8] Morris ZS, Wooding S, Grant J (2011). The answer is 17 years, what is the question: understanding time lags in translational research. J R Soc Med.

[9] Chan TM, Stehman C, Gottlieb M, Thoma B (2020). A short history of free open access medical education. The past, present, and future. ATS Sch.

[10] Cadogan M, Thoma B, Chan TM, Lin M (2014). Free Open Access Meducation (FOAM): the rise of emergency medicine and critical care blogs and podcasts (2002-2013). Emerg Med J.

[11] Cadogan M, Nickson C (2025). FOAM - free open access meducation. https://litfl.com/foam/.

[12] Thoma B, Murray H, Huang SY (2018). The impact of social media promotion with infographics and podcasts on research dissemination and readership. CJEM.

[13] Berk J, Trivedi SP, Watto M, Williams P, Centor R (2020). Medical education podcasts: where we are and questions unanswered. J Gen Intern Med.

[14] Ibrahim AM, Lillemoe KD, Klingensmith ME, Dimick JB (2017). Visual abstracts to disseminate research on social media: a prospective, case-control crossover study. Ann Surg.

[15] Patocka C, Lin M, Voros J, Chan T (2018). Point-of-care resource use in the emergency department: a developmental model. AEM Educ Train.

[16] Thoma B, Chan T, Desouza N, Lin M (2015). Implementing peer review at an emergency medicine blog: bridging the gap between educators and clinical experts. CJEM.

[17] Sidalak D, Purdy E, Luckett-Gatopoulos S, Murray H, Thoma B, Chan TM (2017). Coached peer review: developing the next generation of authors. Acad Med.

[18] Carley S, Beardsell I, May N (2018). Social-media-enabled learning in emergency medicine: a case study of the growth, engagement and impact of a free open access medical education blog. Postgrad Med J.

[19] Azim A, Beck-Esmay J, Chan TM (2018). Editorial processes in free open access medical educational (FOAM) resources. AEM Educ Train.

[20] Grock A, Bhalerao A, Chan TM, Thoma B, Wescott AB, Trueger NS (2019). Systematic Online Academic Resource (SOAR) review: renal and genitourinary. AEM Educ Train.

[21] Ting DK, Boreskie P, Luckett-Gatopoulos S, Gysel L, Lanktree MB, Chan TM (2020). Quality appraisal and assurance techniques for free open access medical education (FOAM) resources: a rapid review. Semin Nephrol.

[22] Raine T, Thoma B, Chan TM, Lin M (2015). FOAMSearch.net: a custom search engine for emergency medicine and critical care. Emerg Med Australas.

[23] Stuntz R, Clontz R (2016). An evaluation of emergency medicine core content covered by free open access medical education resources. Ann Emerg Med.

[24] Gautret P, Lagier JC, Parola P (2020). RETRACTED: hydroxychloroquine and azithromycin as a treatment of COVID-19: results of an open-label non-randomized clinical trial. Int J Antimicrob Agents.

[25] Rochwerg B, Parke R, Murthy S (2020). Misinformation during the coronavirus disease 2019 outbreak: how knowledge emerges from noise. Crit Care Explor.

[26] Mehra MR, Ruschitzka F, Patel AN (2020). Retraction-hydroxychloroquine or chloroquine with or without a macrolide for treatment of COVID-19: a multinational registry analysis. Lancet.

[27] Ioannidis JP (2005). Why most published research findings are false. PLoS Med.

[28] Chan TM, Dzara K, Dimeo SP, Bhalerao A, Maggio LA (2020). Social media in knowledge translation and education for physicians and trainees: a scoping review. Perspect Med Educ.

[29] Paterson QS, Thoma B, Milne WK, Lin M, Chan TM (2015). A systematic review and qualitative analysis to determine quality indicators forhealth professions education blogs and podcasts. J Grad Med Educ.

[30] Lin M, Thoma B, Trueger NS, Ankel F, Sherbino J, Chan T (2015). Quality indicators for blogs and podcasts used in medical education: modified Delphi consensus recommendations by an international cohort of health professions educators. Postgrad Med J.

[31] Colmers IN, Paterson QS, Lin M, Thoma B, Chan TM (2015). The quality checklists for medical education blogs and podcasts. Winnower.

[32] Alyusuf RH, Prasad K, Abdel Satir AM, Abalkhail AA, Arora RK (2013). Development and validation of a tool to evaluate the quality of medical education websites in pathology. J Pathol Inform.

[33] Yang N, Hosseini S, Mascarella MA, Young M, Posel N, Fung K, Nguyen LH (2017). Identifying high quality medical education websites in Otolaryngology: a guide for medical students and residents. J Otolaryngol Head Neck Surg.

[34] Wolbrink TA, Rubin L, Burns JP, Markovitz B (2019). The top ten websites in critical care medicine education today. J Intensive Care Med.

[35] Niforatos JD, Lin L, Narang J (2019). Financial conflicts of interest among emergency medicine contributors on free open access medical education (FOAMed). Acad Emerg Med.

[36] Lin M, Joshi N, Grock A (2016). Approved instructional resources series: a national initiative to identify quality emergency medicine blog and podcast content for resident education. J Grad Med Educ.

[37] Chan TM, Grock A, Paddock M, Kulasegaram K, Yarris LM, Lin M (2016). Examining reliability and validity of an online score (ALiEM AIR) for rating free open access medical education resources. Ann Emerg Med.

[38] Grock A, Jordan J, Zaver F (2021). The revised Approved Instructional Resources score: an improved quality evaluation tool for online educational resources. AEM Educ Train.

[39] Chan TM, Thoma B, Krishnan K, Lin M, Carpenter CR, Astin M, Kulasegaram K (2016). Derivation of two critical appraisal scores for trainees to evaluate online educational resources: a METRIQ study. West J Emerg Med.

[40] Thoma B, Chan TM, Kapur P (2018). The Social Media Index as an Indicator of Quality for Emergency Medicine Blogs: A METRIQ Study. Ann Emerg Med.

[41] Colmers-Gray IN, Krishnan K, Chan TM (2019). The revised METRIQ score: a quality evaluation tool for online educational resources. AEM Educ Train.

[42] Krishnan K, Thoma B, Trueger NS, Lin M, Chan TM (2017). Gestalt assessment of online educational resources may not be sufficiently reliable and consistent. Perspect Med Educ.

[43] Thoma B, Sebok-Syer SS, Krishnan K (2017). Individual Gestalt is unreliable for the evaluation of quality in medical education blogs: a METRIQ study. Ann Emerg Med.

[44] Thoma B, Sanders JL, Lin M, Paterson QS, Steeg J, Chan TM (2015). The social media index: measuring the impact of emergency medicine and critical care websites. West J Emerg Med.

[45] Woods JM, Chan TM, Roland D, Riddell J, Tagg A, Thoma B (2020). Evaluating the reliability of Gestalt quality ratings of medical education podcasts: a METRIQ study. Perspect Med Educ.

[46] Thoma B, Sebok-Syer SS, Colmers-Gray I (2018). Quality evaluation scores are no more reliable than Gestalt in evaluating the quality of emergency medicine blogs: a METRIQ study. Teach Learn Med.

[47] Lin M, Phipps M, Chan TM (2023). Digital impact factor: a quality index for educational blogs and podcasts in emergency medicine and critical care. Ann Emerg Med.

[48] Mayer RE (2002). Multimedia learning. Psychology of Learning and Motivation.

[49] Issa N, Schuller M, Santacaterina S, Shapiro M, Wang E, Mayer RE, DaRosa DA (2011). Applying multimedia design principles enhances learning in medical education. Med Educ.

[50] Issa N, Mayer RE, Schuller M, Wang E, Shapiro MB, DaRosa DA (2013). Teaching for understanding in medical classrooms using multimedia design principles. Med Educ.

[51] Bonk CJ, Lee MM (2020). Motivations, achievements, and challenges of self-directed informal learners in open educational environments and MOOCs. J Learn Dev.

[52] Kim D, Lee I-H, Park J-H (2019). Latent class analysis of non-formal learners’ self-directed learning patterns in open educational resource repositories. Br J Educ Technol.

[53] da Silva AC, Freire FMP, Mourão VHM, da Cruz MD de O, da Rocha HV (2025). Portability and usability of open educational resources on mobile devices: a study in the context of Brazilian educational portals and android-based devices. International Association for Development of the Information Society.

[54] (2025). An approach to undifferentiated shock and hypotension. https://canadiem.org/approach-to-undifferentiated-shock-and-hypotension/.

[55] (2025). PulmCrit- tranexamic acid for traumatic brain injury (CRASH3). https://emcrit.org/pulmcrit/crash3/.

[56] (2019). Effects of tranexamic acid on death, disability, vascular occlusive events and other morbidities in patients with acute traumatic brain injury (CRASH-3): a randomised, placebo-controlled trial. Lancet.

